# “Being Talked to Like I Was a Sex Toy, Like Being Transgender Was Simply for the Enjoyment of Someone Else”: Fetishization and Sexualization of Transgender and Nonbinary Individuals

**DOI:** 10.1007/s10508-021-01935-8

**Published:** 2021-03-24

**Authors:** Annalisa Anzani, Louis Lindley, Giacomo Tognasso, M. Paz Galupo, Antonio Prunas

**Affiliations:** 1 Department of Psychology, University of Milano–Bicocca, Edificio U6, Piazza dell’Ateneo Nuovo 1, 20126 Milano, Italy; 2 Department of Counseling Psychology, Univeristy of Wisconsin–Madison, Madison, WI USA; 3 Department of Psychology, Towson University, Towson, MD USA

**Keywords:** Transgender, Nonbinary, Fetishization, Sexualization, Gender minority stress

## Abstract

Despite the growing interest in the experiences of transgender individuals, the phenomenon of fetishization of transgender bodies and identities has been overlooked. The present study was aimed at investigating the experiences of fetishization of transgender and nonbinary (TGNB) people. Participants in the current study represent a sample of 142 TGNB volunteers from the community who answered the prompt: “If you feel comfortable, could you describe your experience of being fetishized?” Using thematic analysis, we developed three overarching themes relevant to the experiences of fetishization of TGNB participants: (1) context of fetishization; (2) negative experiences of fetishization; and (3) positive or ambiguous experiences of fetishization. The results demonstrated that, in most cases, fetishization was understood by TGNB people as a negative experience of sexual objectification, although some individuals experienced fetishization as a positive experience, perceiving the sexual desire of the other person or living it as a kink. Consistent with the integrated theory of dehumanization, the results demonstrated that both sexual objectification and minority stress contributed to participants’ understanding of fetishization for TGNB individuals. Implications for clinical work with TGNB individuals are discussed.

## Introduction

Despite the growing interest in the experiences of transgender individuals, the phenomenon of fetishization of transgender bodies and identities has been overlooked. The present study investigates transgender and nonbinary (TGNB) individuals’ experiences of fetishization. Fetishization of TGNB individuals is best understood at the intersection of the literature on TGNB attraction and the history of stigmatization of TGNB identities and sexuality.

The term fetishism has a complex history. Its origins date back to colonialism when it was used to refer to pagan cult objects of African cultures (for an in-depth review, see Iacono, 2016). The term has evolved over time and was adopted in psychology and psychoanalysis. Within Freudian tradition, fetishism refers to the investment of a non-genital object to achieve sexual gratification, where erotic attraction is conferred to an inanimate object or a body part not typically considered erotic (Iacono, 2016). From its etymological origin, the term fetishism maintained a negative connotation as a pathological attraction for non-erotic objects. However, within some gay cultures, fetishism has since been assimilated in a depathologizing framework. Within some gay cultures, fetishism is intended as a creative transformation of objects that do not have a primary psychic value for the purpose of sexual pleasure (Amin, 2017). Thus, an object devoid of any personal value is transformed into something to which an emotional value is attributed; in this case, it is linked to sexual pleasure. The argument in support of this new vision of fetishism is noteworthy, because the fetishistic investment of an object is compared to the erotic investment of the genitals, arguing that there is no automatic erotic investment for genitals either, but that this also follows a psychic elaboration.

This reclamation of fetishism within some gay cultures is not universally shared within the broader lesbian, gay, bisexual, transgender, queer (LGBTQ) culture. For example, when fetishism is utilized in reference to an attraction toward transgender individuals, fetishism refers specifically to the sexual investment in transness (i.e., body, identity, status, etc.) as an overvalued sexual object, rather than the holistic individual (Evangelista, 2018). While the literature that has dealt with fetishism of transgender people is scarce, the perspective adopted is mainly focused on fetishization seen as a form of sexual objectification (or sexualization) of transgender women or a sexual attraction to transgender identities (Evangelista, 2018; Flores et al., 2018; Serano, 2007). In the present work, we broaden the conceptualization. On the one hand, fetishization will be understood in a negative sense as a form of sexualization, ultimately dehumanization, of transgender individuals. On the other hand, we will examine fetishization in its more positive sense of being the object of sexual desire and attraction.

### Fetishism as Sexual Objectification

Moradi (2013) suggests that there is overlap between the theories of objectification (Fredrickson & Roberts, 1997), and the theories of stigma and discrimination (i.e., minority stress theory; Meyer, 2003). Objectification theory posits that objectifying a person means to consider them as an object, a mere instrument for the attainment of a personal goal, and which leads to their denial of human dignity. Thus, objectification can be considered a form of dehumanization (Vaes, Loughnan, & Puvia, 2014). Objectification can affect different minorities and social categories, just as different dimensions of identity can be objectified. When these dimensions correspond to the body, where the value of a person is established primarily on the basis of their physical appearance, we refer to it as sexual objectification or sexualization (Fredrikson & Roberts, 1997). Sexualization occurs when someone is reduced to their body parts or sexual functioning (Flores et al., 2018; Fredrickson & Roberts, 1997). Minority stress theory explains the higher levels of psychological distress found in marginalized groups due to the daily stressors and discrimination they are exposed to because of their minority identity (Meyer, 2003). This model, as applied to gender minorities (Hendricks & Testa, 2012; Timmins, Rimes, & Rahman, 2017), recognizes the effects of distal (i.e., episodes of overt discrimination) and proximal (i.e., internalized transphobia) stressors in determining the health status of TGNB individuals (Lindley & Galupo, 2020; Rood et al., 2016, 2017a, b).

Moradi (2013) proposed a pantheoretical model of dehumanization, in an attempt to integrate theories of stigma and objectification with gender minority stress theory. On the one hand, the experiences of discrimination and body objectification both start from a dehumanizing perception of the target. Therefore, both experiences come from the same matrix of dehumanization, and discrimination can be considered a form of dehumanization (Moradi, 2013). Moreover, the specific stressors faced by a given target group may intersect with dehumanization factors in determining the health consequences for minorities. Two studies to date applied the pantheoretical model of dehumanization to TGNB individuals (Brewster, Velez, Breslow, & Geiger, 2019; Velez, Breslow, Brewster, Cox, & Foster, 2016). The first study by Velez tested the effects of constructs of objectification theory (i.e., internalization of social standards on attractiveness) and gender minority stress factors on compulsive exercise in transgender men. The authors highlighted how the variables related to objectification account for part of the negative behavior outcomes, in this case compulsive exercise, and the specific factors of gender minority stress partially explain such relations (Velez et al., 2016). Similarly, Brewster et al. investigated the effects of objectification and gender minority stress on the mental health of transgender women. In Brewster et al.’s model, the dehumanization factors (gender minority stress and objectification) explain the negative outcomes in terms of eating disorders, body surveillance, and body dissatisfaction in transgender women (Brewster et al., 2019). Thus, these studies have shown that the theories of objectification and gender minority stress are not independent; instead, specific factors of both theories work together to determine negative health outcomes in TGNB individuals.

Serano (2007) describes the sexual objectification of transgender women and demonstrates how minority stress and objectivation interact to oppress transgender women, although Serano would herself would not claim to be working from such a model. Serano distinguishes between being the object of sexual desire, something that many people aspire to, and being sexualized. Being the object of desire for others can serve both short- and long-term goals, including the attainment of sexual satisfaction and pleasure, or building a lasting relationship with another person (Buss, 1998; Zawacki et al., 2009). Sexualization, in contrast, is aimed at establishing a power dynamic, subjecting transgender (and cisgender) women to cissexist power (Serano, 2007). Serano gives many examples of how transgender women are sexualized: from commentary and catcalling, to the sexualized images passed through media, and to the role played by transgender women in porn. Continuous exposure to these experiences and media images reinforces the gender roles that see men as predators and (transgender and cisgender) women as prey. Serano distinguished between sexualizing experiences of when she is perceived as a transgender woman and as cisgender woman. When perceived as the former, Serano reports far more intrusive experiences, such as male strangers immediately engaging in a conversation about their sexual fantasies or sexual desires. According to the author, the sexualization of transgender women stems from an erroneous, basic assumption: that transgender women undertake a transition journey in order to be more attractive to (heterosexual cisgender) men. For example, this assumption assumes transgender women undergo gender-affirming surgery, such as vaginoplasty, in order to facilitate insertive sex with men and not to affirm their identity. This perspective deprives men of responsibility for their inappropriate and unsolicited actions and blames transgender women who “had it coming” (Serano, 2007). Therefore, in this power dynamic, the bodies of transgender women are reduced to being sexual objects for men. Serano’s work is specific to the experiences of transwomen, as she does not extend the argument to transmasculine and nonbinary individuals.

The literature on objectification and self-objectification has predominantly focused on cisgender women, who adhere to a system of beliefs of benevolent sexism by internalizing the objectifying male gaze (Calogero, 2011). Being the object of sexualization can provoke positive emotions in women when they comply with the rules of clothing, appearance, and behavior because of the advantages that can follow (Smolak & Murnen, 2011). The literature also mentions self-objectification by transgender women including body shame, increased self-monitoring, and anxiety (Sevelius, 2013). In particular, Sevelius describes sexual objectification as a form of psychological distress experienced in relation to stigma. Sexual objectification exposes transgender women to greater risk, particularly in sexual situations. Self-objectifications among other gender minorities (i.e., transmasculine and nonbinary individuals) is not equally explored in literature.

### Fetishism as Sexual Attraction

Despite the paucity of literature on the sexual attraction aspect of fetishization of TGNB individuals, the existing literature represents TGNB individuals as lacking sexual self-confidence and attraction to TGNB individuals as pathological. As noted above the consequences of being discriminated against are well explained by the gender minority stress model and have an impact at the individual level and interpersonal level (Hendricks & Testa, 2012). For TGNB individuals, in fact, internalizing negative beliefs about one's gender identity can have a negative impact on their self-esteem (Austin & Goodman, 2017). In terms of romantic and sexual relationships, this could result in feeling less desirable and attractive in the eyes of others (Lindley, Anzani, Prunas, & Galupo, 2020), as well as being hypervigilant or suspicious toward others (Rood et al., 2016). Additionally, sexual desirability might be influenced by cisgenderist conceptualizations of sex, which norms sexuality and sexual practices, for example focusing on insertive sex (Blumer, Ansara, & Watson, 2013). Another implication is lack of sexual assertiveness, for example, the assumption of being undesirable of TGNB may lead them to show gratitude whenever they become the object of sexual interest from others (Bartholomaeus & Riggs, 2017). This might expose them to violence or sexually risky behaviors as found within the literature on transgender women which highlights sexual objectification, dehumanization, as well as aspects of fetishization in the context of intra-partner violence (Barrett & Sheridan, 2017; Flores et al., 2018; Serano, 2007).

Tompkins’ (2014) work on the narrative of the “tranny chasers” starts from a completely different premise. Using a digital ethnographic methodology, analyzed data from blogs and YouTube videos of White cisgender women partnered with transmasculine individuals. Tompkins argued that the discourse around fetishizing TGNB people is limited and inherently pathologizes desire and attraction toward TGNB individuals, whether this is in relation to specific partners or a preference for dating TGNB people in general. Thus, the rhetoric of transfetishism may prevent cisgender individuals from talking openly about their attraction to TGNB people in public spaces and online, making it a taboo (Tompkins, 2014). This analysis did not examine directly the experience of TGNB individuals, but instead centered the experience of cisgender partners. Tompkins’ research explores potential positive aspects of fetishism for transgender people (i.e., emphasizing the aspects of attraction and sexual desire in a positive way). This perspective on healthy sexual and romantic relationships could prove to be an important factor of protection from the negative effects of discrimination, as already reported in the literature (Galupo, Pulice-Farrow, Clements, & Morris, 2019; Meier, Sharp, Michonski, Babcock, & Fitzgerald, 2013; Pulice-Farrow, Bravo, & Galupo, 2019).

### Current Study

Given the complexity of the variables involved in the fetishization of transgender individuals, the present research frames fetishization broadly. We understand fetishism both as a form of sexual objectification and as a construct that refers to sexual desire and attraction. Through this framework, the present work centers the experiences of TGNB individuals in order to understand individual perceptions and experiences of fetishization. The study aims to fill a literature gap that only a few contributions have addressed so far. In particular, our focus will include a range of different transgender and nonbinary identities, abandoning the idea that this is an issue that concerns only transgender women. Thematic analysis was used to address the following research question: In what ways do transgender individuals describe their experiences with fetishization?

## Method

### Participants

Participants were recruited through flyers posted on various online resources that served the transgender community specifically, as well as the LGBTQ community generally. The recruitment flyer revealed the purpose of the study, inclusion criteria, as well as the contact information of the primary investigator. The present research is part of a broader project on the sexual health of TGNB individuals. Out of the total number of respondents to the broader investigation on TGNB sexuality (*N* = 466), 64.2% (*n* = 299) of participants answered “yes” (*n* = 177, 38.0%) or “maybe” (*n* = 122, 26.2%) to the question: “In your experience, have you ever felt fetishized?” Of these respondents, 142 participants provided a response to an open-ended question regarding their experiences with fetishization. The current study was focused on those participants who responded to this open-ended prompt.

The age range of participants varied from 18 to 64, with the majority of participants in the age range of 18–34 years old. All participants were English speakers and participation was not restricted to any specific geographical location (see Appendix 1 for a map representing the geographic location of the respondents). The sample has limited racial/ethnic diversity, with 72.5% of the participants identifying as White (*n* = 103) and 27.5% (*n* = 38) as a marginalized racial/ethnic identity. Full sample demographics can be found in Table 1.

**Table 1:** Demographic characteristics of the sample

Age (in years)	*N *(%)
18–24	60 (42.3)
25–34	46 (32.4)
35–44	28 (19.7)
45–54	4 (2.8)
55–64	4 (2.8)
Gender identity	
Transfeminine	34 (23.9)
Transmasculine	59 (41.5)
Nonbinary	42 (29.6)
Agender	7 (4.9)
Sexual identity	
Asexual	10 (7)
Bisexual	35 (24.6)
Fluid	1 (0.7)
Gay	14 (9.9)
Heterosexual	8 (5.6)
Lesbian	7 (4.9)
Pansexual	25 (17.6)
Queer	29 (20.4)
Other	13 (9.2)
Race/ethnicity	
American Indian/Alaska Native	1 (0.7%)
Asian/Asian American	2 (1.4)
Black/African American	3 (2.1)
Hispanic/Latin	10 (7)
White	103 (72.5)
Biracial/Multiracial	7 (4.9)
No answer	6 (4.2)
Other	10 (7)
Education level	
Less than high school	2 (1.4)
High School graduate	45 (31.7)
2 year degree	19 (13.4)
4 year degree	44 (31)
Professional degree	25 (17.6)
Doctorate	7 (4.9)
Relationship status	
Married	16 (11.3)
Committed	32 (22.5)
Dating	22 (15.5)
Open relationship(s)	19 (13.4)
Single	45 (31.7)
Not interested in having a relationship	8 (5.6)

### Measures and Procedure

This study focussed on the description of episodes of fetishization experienced by TGNB people. Participants completed an online survey where they answered questions regarding their demographics and experiences with sexually related activities. Demographic questions included highest level of education, relationship status and style, and race/ethnicity. As part of the demographics, participants provided their gender and sexual identity as a write-in response as well as selected fixed, static, identity choices. As this study is part of a larger project investigating the sexual health of transgender individuals, participants were then asked to complete several quantitative measures that assessed sexual satisfaction, body satisfaction, gender dysphoria, experiences with fetishization, and transition steps undertaken. Participants who indicated “yes” or “maybe” to the question: “In your experience, have you ever felt fetishized?” were then asked to indicate in which situations they felt fetishized (e.g., on social media, on dating apps, interpersonally, or in other contexts). Finally, participants were asked to describe their specific experience of fetishization through answering the following prompt: “If you feel comfortable, could you describe your experience of being fetishized?” Participants were not provided with an operational definition of fetishization. This methodological choice was based on the fact that we wanted participants to be able to give a free interpretation of their experience of fetishization without necessarily characterizing it in a negative or positive way. Participants were able to skip any item they did not wish to answer, including both quantitative measures as well as qualitative questions, and were not limited in the length of response.

The study was approved by the Institutional Review Board at Towson University. Participants filled out an online survey investigating sexuality of TGNB individuals. There was no incentive provided for participation. Once the participants finished the survey, we thanked them for their participation and provided them with an opportunity to leave suggestions to improve future studies.

### Analysis

Thematic analysis was utilized to evaluate the qualitative descriptions of episodes of fetishization experienced by TGNB participants (Braun & Clarke, 2019). The coding team (consisting of the first, third, and last authors) began the data analysis process by independently identifying potential themes of fetishization included in the participants’ descriptions of episodes of fetishization. During the coding process, the coding team met several times to discuss potential themes, review their observations, and agree upon an initial set of themes. The team independently coded the data set into the agreed upon thematic structure. Participant identities were masked during coded. The coding team met three times to finalize the coding scheme. Final coding resulted in an inter-rater reliability that ranged from 85% (Fleiss’ *k* = 0.85) to 96% (Fleiss’ *k* = 0.96). Discrepancies in the categorization of participants’ descriptions were resolved via discussion, and final results were unanimously agreed by all the members of the coding team.

#### Positionality

The research team included a postdoc researcher who identifies as a White Italian cisgender queer woman, a first year Ph.D. student in Counseling Psychology who identifies as a White queer transgender man, a clinical psychologist who identifies as a White Italian cisgender heterosexual man, a Professor of Psychology who identifies as a biracial agender pansexual person, and a Professor of Psychology who identifies as White Italian cisgender gay man. The coding team was represented by the Italian authors of the research group, with the first and last/senior author being experts in gender identity research. An Italian transgender consultant read the final coding scheme to assess whether it could capture all the nuances of the TGNB research participants' experience. The coding team was supervised by the American group (2nd and 4th authors). The diversity of experience of the authors for gender, sexuality, and race/ethnicity allowed us to approach our coding discussions from different perspectives.

## Results and Discussion

### Descriptive Statistics on the Occurrence Fetishization

Of the total number of respondents to the broader investigation on TGNB sexuality (*N* = 466), 64.2% (*n* = 299) of participants answered “yes” (*n* = 177; 38%) or “maybe” (*n* = 122; 26.2%) to the question: “In your experience, have you ever felt fetishized?” Chi-square analysis did not reveal any differences in the distribution of the answers between transmasculine, transfeminine, and nonbinary individuals, χ^2^(4) = 8.02, *p* = .09. In addition, those who reported an experience of fetishization identified the experience within interpersonal interactions (63.5%), on dating apps (53.2%), or on social media (56.9%). About 8.7% of individuals who felt fetishized also mentioned other contexts of fetishization, such as workplace, clubs, regular media, hook-ups, and in the context of sex-work. We also asked about the fear of being fetishized, where 33.1% of respondents reported low/no fear (“definitely [did] not” or “probably [did] not”) of fetishization and 18.7% were neutral (“might or might not”). However, 48.2% of participants reported some fear (“probably yes” or “definitely yes”) of fetishization.

### Qualitative Investigation of TGNB Individuals’ Fetishization Experiences

The thematic analysis revealed eight themes grouped in three overarching categories that represented participants’ experiences of being fetishized: (1) context of fetishization; (2) negative experiences of fetishization; and (3) positive or ambiguous experiences of fetishization. The coding scheme, with theme definitions, is reported in Table 2. The results are described using illustrative quotes accompanied by participant’s ethnicity, self-defined gender identity, and sexual identity. Often the participants' narratives cover more than one theme as the themes were not mutually exclusive. As it is standard for qualitative research, results and discussion are presented in an integrated section (Flick, 2014).

**Table 2:** Coding scheme

Context of fetishization	
Cisgender men	Describing being targeted by gay closeted, gay, straight guys, dudes, etc.
Cisgender women	Describing being targeted by lesbians, cisgender women
Social media and dating apps	Describing the experience of fetishization facilitated by dating apps or social media
Negative experiences of fetishization	
Disgust and distress responses	Describing feeling disgusted and/or distressed
Fear and avoidance	Describing a reaction of fear or avoidance in response to fetishization or anticipating a situation of fetishization
Sexual objectification	Describing to feel like an object, a sexual object or being dehumanized
Microaggressions	Describing situations in which someone denied or invalidated the individual’s gender expression or identity
Positive and ambiguous experiences of fetishization	
Positive	Describing positively the experience of being fetishized
Ambiguous	Describing both positive and negative aspects of the experience of being fetishized

#### Context of Fetishization

TGNB participants frequently described their experiences as involving specific contexts or sources of fetishization. Participants discussed being fetishized by: (1) cisgender men; (2) cisgender women; and (3) on social media and dating apps.

In reporting their experiences, TGNB participants’ descriptions centered the identity of the person who was fetishizing them. In the vast majority of cases, participants cited cisgender men and women as contributing to their experiences of fetishization, with cisgender men being mentioned most often. Some participants mentioned being fetishized by straight men.

This only really happens in situations where I'm out about being trans. And it always seems to be cis het [cisgender heterosexual] men. I think quite a few men are into the idea of a transgender woman who looks cis. (White, female, lesbian)

And a few participants mentioned also being fetishized by gay and bisexual men.

On a dating app for gay men called Scruff, you can tag yourself as being transgender, as well as tag yourself as someone looking for a transgender partner. I used this app and within minutes received messages from self-identified straight men trying to pressure me to meet and have sex with them. (White, transgender man, pansexual)

In some cases, participants described a tension between the sexual identity of the other person and their own gender and sexual identity. In particular, transmasculine participants described being fetishized by men who self-identified as heterosexual. By having sex with transmasculine people, heterosexual men believed they could have sex with other men without disavowing their heterosexual identity. The fact that some transmasculine individuals may have vaginas would, according to their interpretation, be the justification that allows them to still call themselves heterosexual.

I've felt fetishized in a variety of ways, but especially by bicurious or closeted men who view transgender men as either a “safe” way to engage in sex with another man (while still retaining some plausible deniability: their claim to heterosexuality), as a way to fulfill a fantasy of having sex with a lesbian or as a way to deny their own non-heterosexual attraction. I have even been pursued by men who felt that, as a non-T post-top-surgery transgender man, I was similar to a prepubescent boy, and found this attractive. (White, transgender male, gay)

The literature emphasizes that sexual behavior, sexual attraction, and sexual identity do not always coincide (Geary et al., 2018). With regard to their own sexual identity label, TGNB individuals use gender identity (versus coercively assigned sex or specific body parts) to define their own sexuality (Galupo, Henise, & Mercer, 2016). It makes sense, then, that transmasculine participants described feeling fetishized by the fact that heterosexual men justified their sexual interest in them by focusing on specific body parts or on specific sexual behaviors thereby discounting their identities as men. This conflict becomes clear in the following example, where the sexual behavior (i.e., having a sexual encounter with a transmasculine person) is assumed to have a direct effect on how a person should define their sexual orientation (i.e., if you have a sexual encounter with a man you should define yourself as a label other than straight). In a way, some TGNB participants seem to invalidate the identity of the other when it conflicts with theirs or does not reinforce it.

Straight men claiming an interest in me, and they dislike it when I tell them that makes them not straight. […] I don't mind people specifically being interested in transgender men, but you aren't straight if you are! (White, male, queer)

Although to a lesser extent, cisgender women have also been reported to be agents of fetishization toward TGNB individuals, mostly by transmasculine individuals. In this sense Tompkins’ (2014) critique about term fetishization can be considered limited and limiting. In fact, many TGNB people experience certain behaviors of cisgender people toward them as dehumanizing and objectifying. The risk of not talking about fetishization would be to deny an experience lived by transgender people. In particular, Tomkins’ discourse referred to the pathologization of the attraction of cisgender women toward transmasculine individuals. However, cisgender women were not excluded from the TGNB participants’ descriptions of fetishization.

There is a habit in the cis lesbian culture of fetishizing transgender men who are still read as lesbians. This changes once transgender men are read as cis male. (White, male, heterosexual)

In the descriptions of fetishization participants also frequently mentioned the context of their experience. TGNB participants often described the online environment as a tool used for fetishization. Social media and dating apps were identified among the main digital tools in which they have felt fetishized. Social media was described in a negative way, either as places where participants received messages of insult or threat, or where participants could read posts with messages of sexual objectification toward TGNB people.

I have repeatedly had to block strangers and mutual friends on social media for messages like, “I bet the right dick could cure you” and “Give me an hour, I could make you a REAL woman.” One creepy old guy referred to me as “a reasonable substitute for gay pedophiles.” (White, nonbinary, demisexual)

Some posts on social media just feel like they don't see me as a person. Just as a way to get clout or as a more appealing sex object because I'm assumed to be more feminine and possibly to still have a vagina, but have an otherwise male appearance. (White, man, asexual)

As for dating apps, the situation becomes even more explicit and has more sexual connotations. Participants reported that they have received invasive questions and unsolicited sex requests.

I have never felt fetishized when talking to women. However, when talking to men on apps like Grindr, it is generally an unpleasant experience. It's mostly older white men who are clearly interested in transgender men because it's “exotic” or even because we sometimes look underage… (White, male, queer)

In addition, there are online services which are made especially for fetish, where TGNB people willingly make themselves available to be the subject of fetishization, as in the following example:

On the website Fetlife, I have been approached often because I’ve listed myself as FTM and it’s pretty obvious it’s a kink. (White, transgender male, queer)

In the TGNB participants' descriptions, the context that emerged predominantly as fetishizing was the online environment. However, it is also worth mentioning the sex work context. This emerged in a more tangential way, such that it could not represent a theme in itself, but it is worth mentioning.

[…] Having a man constantly talk about my penis and how "great" it is while I was naked. This final instance is the only case in which I was working as a sex worker. (White, nonbinary transgender girl, lesbian)

The case below is particular in this respect. It reports the experience of a transfeminine person who resigned herself to be the “waste bin object” of one night's desire after repeated experiences of fetishization. She no longer imagines that she can arouse the authentic interest of someone for herself as a person, she has internalized the vision of herself as a sexual object. Sex work is seen almost as a way of making money out of a situation that has a very high cost for her.

[…]So eventually you start to just settle for being the fetish. You start settling for being a waste bin object of desire for a night. Eventually, you realize you may as well get paid for your troubles. I'm starting to see why all my sisters are in the sex trade now… (White, female, heterosexual)

Other contexts of fetishization have been mentioned by single individuals and concern the fetish connected to the world of erotic narrative or porn.

In porn and erotic works that depict gay transgender men, AFAB [assigned female at birth] language in reference to genitalia is often used. This triggers my dysphoria and feels very gross. (Unknown, transgender man, gay)

It is important to note that the context for fetishization (relational vs social media) is relevant in terms of the impact. A single, negative experience of online fetishization differs from a context in which the experience is more frequently repeated, such as within a relationship. Negative experiences (e.g., rejection) suffered by TGNB individuals within a romantic relationship have been linked to increasing levels of anxiety and depression; on the contrary support in romantic relationships is related to lower levels of psychological distress (Fuller & Riggs, 2019). Thus, it is likely that fetishization and sexual objectification may be experienced as rejection within a relationship. The relational impact may also be magnified as it could be experienced as a disruption of social support (Pulice-Farrow, Brown, & Galupo, 2017).

Another important note on the context of social media and dating platforms is the intent of the TGNB individual when engaging with them. For example, individuals who use platforms dedicated to specific kink may interpret fetishization in a more positive way and not as a form of sexual objectification but rather as form of sexual desire. Whereas individuals who use apps to seek friends or romantic partners but instead receive messages of sexual objectification may be harmed by the same experience. Thus, social media and dating platforms can be viewed as a context in which the individual can be fetishized but the effects on wellbeing may be buffered by the individual’s goal when engaging (i.e., kink or intimate connection).

Overall within the context category, participants most often described cisgender men and women as agents of fetishization, with the fetishization taking place within specific contexts, the most frequent being that of dating apps and social networks. Sex work and porn were also mentioned as environments in which TGNB people felt sexually objectified.

### Negative Experiences of Fetishization

When asked about their experiences of being fetishized, TGNB participants described feelings of being used as sexual objects, dehumanized, or invalidated. These descriptions encompassed an overall experience in which their individual value as human beings were demeaned. Four main themes emerged as negative experiences of fetishization: (1) disgust and distress responses; (2) fear and avoidance; (3) sexual objectification; and (4) microaggressions.

The negative reactions to being the object of sexual objectification and fetishization for many participants often corresponded to an emotional feeling of nausea and disgust. In the following example, it can be observed that the person who narrates this fetishization experience had a clear understanding of the point at which the line between attraction and fetish is drawn. This line would stand between doing sexual activities together or doing sexual things to the TGNB person, thus sexual objectifying them.

On a vanilla date, one guy told me “I’m the best of both worlds” and wouldn’t stop talking about all the things he wanted to do to me. (Not with me) Just gross. (White, transgender male, queer)

As in the previous example, many narratives include words related to the feeling of disgust, such as gross, sickening, or disgusting.

Some people think being transgender is “hot” which is screwed up, I had a partner (cis male) that once I passed as male broke up with me because I did not look like “a trap” anymore. Disgusting. (White, male, gay)

Other TGNB individuals described more the feelings of distress and discomfort given by the situation of fetishization. Once again, the fine line between attraction and fetishization is clear to participants. The problem is not about attraction to a TGNB person, but when this person is seen only as a sexual object, in a sub-human way.

Not really very comfortable with the feeling. I’d rather be cared for/love for myself, not for what fetish I can fulfill. (Black/African American, male, gay)

Among the negative reactions to the experience of fetishization were responses of fear, avoidance of certain situations that could expose the participant to fetishization, and limitations of individual freedom. In some cases, the avoidance of certain virtual and physical places was linked to vicarious fear developed in response to the stories and experiences of other TGNB people in the community.

I personally have never been fetishized (that I know of) but I stay away from a lot of spaces where it could happen due to fear. I know many other trans/enby [nonbinary] people that have had experiences with fetishization and don't want it to happen to me. This is very limiting to me. (White, male, bisexual)

In other cases, the avoidance of certain situations followed participants’ personal negative experiences.

Gross. If the only interesting thing about me was a penis that shouldn't even be there…that's why I didn't date for the last two years before GCS [gender confirming surgery]. (White, female, heterosexual)

One of the proximal stress factors to which TGNB people are exposed, according to the gender minority stress model, is the expectation of rejection, which in this case becomes the expectation of having a negative experience linked to being the object of sexualization or fetish (Hendricks & Testa, 2012).

TGNB participants described the experience of feeling objectified in various ways. TGNB participants in many cases explicitly mentioned feeling treated as less than human or like sexual objects when describing their experiences of fetishization. In some cases, sexual objectification involved a symbolic fragmentation of the body, which is separated from the rest of the person and considered as a mere instrument for the sexual pleasure of others. This fragmentation can be seen in the following example in which the participant describes themselves as a sex toy, an object for another’s pleasure:

Being talked to like I was a sex toy, like being transgender was simply for the enjoyment of someone else, dehumanized. (White, female, bisexual)

Some participants described feeling as if they were simply a tool for others to use to fulfill a fantasy or to try out a novel sexual experience. These experiences left participants feeling as if they were not seen as real humans or individuals with emotions.

Hooking up using Grindr is risky as a transgender guy as any connection could come at a personal cost. I'm called slurs and/or messaged by men interested in fulfilling a fantasy/experimenting with their own sexuality. Sometimes I feel like, in hooking up with a cisgender guy, I am perceived as a woman or woman-substitute for using my vagina instead of my ass. (White, man, gay)

TGNB participants additionally described being fetishized or used solely for their masculine or feminine appearance.

As a nonbinary person who was AFAB [assigned female at birth] and is masculine-presenting […] Basically, in my experience, it had felt like women were attracted to me because of my appearance and my appearance alone, rather than being attracted to me as a person in addition to my appearance. (White, nonbinary, lesbian)

Some specific features of TGNB bodies seem to be fetishized more often than others. Participants described being approached for their juvenile appearance, “I have had men specifically seek me out on dating apps because of my gender identity, they like the idea of a young-looking boy with a vagina they can push around” (White, transman, pansexual); the coexistence of stereotypical feminine and masculine characteristics in the body, “I have been told I am the perfect partner because I am neither a man nor a woman since I have a vagina, mostly by cisgender men even though I identify as a man” (White, man, gay); some specific body features, “I have large breasts which I feel are fetishized by both males and females” (White, genderfluid, bisexual); and also the identity intersection with their ethnicity; “Racial fetishization as a south Asian AFAB [assigned female at birth] person” (Asian/Asian American, genderqueer, pansexual).

Reducing the body to its sexual function is not the only negative experience reported in the narratives of the TGNB participants’ fetishization experiences. Often participants described fetishization in the form of microaggressions. Microaggressions for transgender individuals often take the form of identity invalidation, delivered through verbal, behavioral, and environmental indignities (Nadal, Davidoff, Davis, & Wong, 2014; Torino, Rivera, Capodilupo, Nadal, & Sue, 2019). Further, transgender individuals often experience identity invalidation in the context of both friendships and romantic or sexual exchanges (Galupo, Henise, & Davis, 2014; Pulice-Farrow et al., 2017). In particular, microaggression may invalidate the individual’s identity, deny or exclude their personal experience, emotions, or thoughts, and often are based on stereotypes. In the context of fetishization, TGNB participants described how others utilized communication to invalidate participants gender identity or to deliver insulting messages. The invalidation of gender identity can occur through misgendering, the use of incorrect pronouns to address the person.

One person made the comment about how he was okay with him losing his virginity to a Trans* person, but still referred to me as “she.” (White, male, gay)

Another way to invalidate gender identity is to label or describe TGNB individuals with a narrative that does not match their own. For example, reducing gender identity, or manhood and womanhood, to the possession of certain physical features or body parts, “I have been told I am the perfect partner because I am neither a man or a woman since I have a vagina, mostly by cisgender men even though I identify as a man” (White, man, gay); or by distorting the gender experienced by an individual, “[…] reducing AFAB transgender people to “confused lesbians” or “butch lesbians” even when many of us have never identified as lesbians and may not even be interested in women” (Middle Eastern, nonbinary transgender guy, asexual)

In some cases, microaggressions in the context of fetishization become actual insults and offenses. The intention is always to diminish the value of the identity of the TGNB person.

Someone wanted me because I’m “the best of both worlds.” They used inappropriate words to describe my gender, trivialized my experience, and saw me as a potential “fun experiment.” (White, nonbinary, bisexual)

In other cases, the negative effect of microaggression is that the TGNB participant felt turned off during sexual or romantic encounters.

Not sure if fetishized alone or in combination with a strong feeling of being misgendered. Experiences with cis men and cis women who read me as a “type” of “woman” they liked which invalidates their attraction and any of our interactions in my eyes as I don’t identify as a woman. (White, agender, queer)

The microaggressions described are often supported by stereotypical assumptions. The assumptions, which uphold the microaggressive messages in the context of fetishization, can refer to the sexuality of TGNB people. With regard to sexuality, some TGNB individuals described receiving sexual requests made because of the assumption about what TGNB people might like or enjoy. In other words, some people expect TGNB individuals to do “certain things” in sex. For example, transgender women are considered to be hypersexual, “As a transgender woman, I’ve felt guys think I’m only into sex and particularly sex that fits a fetish” (White, transgender female, heterosexual); nonbinary individuals mentioned that their transgender status is stereotypically associated with the idea that they have multiple sexual partners at a time and enjoy group sex, “Being transgender supposedly meaning I am inherently into polyamory, group sex…” (White, nonbinary trans, bisexual), “I have been approached several times and propositioned for threesomes, in which I have expressed no desire to participate” (Australian Aboriginal, nonbinary, bisexual); and transmasculine individuals mentioned how their identity is linked to a particular sexual role, “I feel like men see me as this short hairless ‘guy’ who would fit this stereotypical submissive bottom role” (Hispanic/Latin, nonbinary, gay), “I am a postoperative phalloplasty person who sometimes uses male dating apps. If I disclose that I have a penis, men are not [interested]in me. They expect me to have a front hole that I want to use” (Black/African American, male, bisexual).

In a few cases, fetishization experiences were described as going far beyond microaggressions, resulting in violence and abuse.

I hate it. It feels like the second people find out that I am transgender they see me only as a thing to be fucked, and not in a good way. Many understand that they can rape me with no legal recourse, unlike cis-women. (American Indian/Alaska Native, female, bisexual)

Although the questionnaire did not explicitly ask about experiences of violence or abuse, it is important to note that they were spontaneously reported by individuals who self-identify as transgender women. As also denounced by Serano (2007), it is possible that a dynamic of power over transgender women’s bodies and their sexuality exposes them to a higher risk of violence and abuse (James et al., 2016).

To summarize, it seems that the context of fetishization certainly allows for the sexualization of the TGNB individuals’ body and identities, but it also gives rise to microaggressions that deny the experience of the TGNB individuals or demean their identity. In this particular context of fetishization, microaggressions often take the form of attacks based on a person’s appearance, on their possession (or lack thereof) of certain body parts, and on stereotypes and assumptions related to sexuality. In this sense, microaggressions are very specific and might be considered a particular vehicle for the fetishization of transgender individuals.

#### Positive or Ambiguous Experiences of Fetishization

The third category of themes reflects TGNB participants’ overall positive or ambiguous experiences of being fetishized. Participants often described the episode in which they were subjected to fetishized sexual attention in positive or ambiguous ways. For a few participants, the experience of fetish was framed as a part of their kink sexual practice and thus they viewed the experience as positive.

I'm into kink so it's usually fine. I do get upset about straight men fetishizing lesbians. (White, nonbinary, pansexual)

One participant described the experience as positive because it allowed them to have more sexual attention than before.

I love knowing that other femboys are masturbating to me, just like I masturbate to them. Honestly, I don't understand what's bad about being fetishized, because it's a million times better than when I was a normal straight guy who no women ever showed any sexual interest at all. (At worst I’m averagely attractive because I'm not fat, not ugly, and I have a full head of hair) (White, femboy, heterosexual)

Another participant described enjoying the extra sexual attention of men when presented as feminine.

When I crossdress I get a lot of sexual attention from men, and I enjoy it. (Hispanic/Latin, male, fluid)

Finally, a transgender woman described fetishization as a positive experience, specifically because her interactions were not limited to sexual objectification as a transgender person; rather the others’ interest in her eventually extended to her as a whole person.

I was among a safe space where fetishization was not the end of their interest in me as a person. Yes, I was “the transgender girl” but they in later interactions were respectful of me as a person. Regarding the next question, my experiences would be an exception. (White, female, bisexual)

While few participants described their experiences with fetishization as positive, some more TGNB participants described fetishization as having an ambiguous value (e.g., both positive and negative). On the one hand, being fetishized allowed TGNB individuals to experience being the focus of sexual attention and interest. On the other hand, it reduces TGNB individuals to their sexual appeal only, with other characteristics being ignored. This dichotomy is exemplified in the following participant response:

When having practically no option for feeling desired, being fetishized seems better than having no attention at all. (White, man, bisexual)

Some participants described the feeling of ambiguity as the tension between having attention and feeling dehumanized and used.

Someone is interested in me and I usually feel good and wanted, until they make a comment about me that I do not like- they like boycunt or FTMs or they think I’m the best of both worlds or whatever. Then I realize they’re fetishizing me and I feel gross. (White, genderqueer, White)

In other cases, the fetishization passes through the invalidation of identity, and individuals may realize they have been sexualized and their value reduced to having (or not having) certain sexual characteristics or body parts.

When I came out to a gay cis guy (just after I came out to myself) I was seeing casually, he said he didn’t care about gender and just that I didn’t have boobs or a vagina. On one level accepting, on the other kind of reductive. (Ashkenazi Jewish, genderfluid, bisexual)

Some TGNB participants compared the experience of being a fetishized to the experience of discrimination. Compared to episodes of transphobic aggression or microaggression, fetishization for these participants took on a positive connotation.

I've encountered more transphobia than fetishizing so it makes the fetishizing seem welcome by comparison. (White, FtM, gay)

As one of the participants pointed out, “There is a fine and perceptible line between being her type and being her fetish” (White, genderqueer, queer). When TGNB participants feel that the line is crossed (i.e., they feel that they are not the object of attention as people with personal identity and history, but they are perceived as sexual objects) their perception of fetishization becomes negative.

### Conclusions

The present findings emphasize transgender fetishization as taking the form of both objectification of transgender bodies and attraction to transgender identities. Consistent with the integrated theory of dehumanization (Moradi, 2013), the results of the study demonstrated that for most participants both sexual objectification and minority stress contributed to participants’ understanding of fetishization for TGNB individuals. The results highlight how TGNB individuals’ experiences of fetishization were frequently described as objectifying and dehumanizing, leading them to feel as if they are not being seen as real people but merely sexual objects. It is worth noting that we did not provide an a priori definition of fetishization. Instead, participants’ responses reflect a broader definition and capture elements that have been overlooked in previous research on fetishization. Thus, we captured TGNB participants’ narratives who had positive experiences of fetishization.

The positive accounts offered by a few participants could be interpreted as the internalization of the self-image as sexual objects. While this concept of self-sexualization is most often reported among transwomen (Sevelius, 2013), within our sample this positive view of themselves as objects of a fetish was experienced by transmen and nonbinary individuals as well. From the narratives of some of the participants, it would seem that fetishization was related to obtaining sexual attention, which, to some extent, had an immediate positive effect on self-esteem. It is also possible that the search for gratification given by sexual appreciation may also have a role of confirmation and validation of one’s gender identity.

Attitudes toward fetishization were polarized between positive and negative reactions, and ambiguous reactions. On the one hand, fetish is seen as a sexual kink or a momentary booster of self-esteem when feeling appreciated. In some cases, this result can be interpreted as self-objectification, in which the TGNB person internalizes the message that their personal value is conveyed by their sexual function or attractiveness. On the other hand, a negative attitude toward fetish includes reactions of fear and avoidance of situations that can lead to sexualization.

Regardless of participants’ positive or negative view of fetishizing experiences, the qualitative results and the descriptive statistics on the frequency of the experience reveals that the experience of fetishization is quite relevant for the TGNB population, involving more than half of the research participants of the current study. The fetishization, besides taking place in interpersonal relationships, is often mediated by online tools such as dating apps and social media. This result has been detected both by descriptive analysis and qualitative responses. Online platforms are configured as a means to send messages or publish posts which were perceived as harmful by some of our TGNB participants. Often these messages contained microaggression which were objectifying and dehumanizing. For example, these messages reduced the participant’s sexual function and value to having (or not having) certain bodily characteristics.

The literature so far has had very little focus on the issue of fetishization and has centered mainly on the perspective of transgender women. It is important to underline that our sample is representative also of transmasculine and nonbinary individuals. Moreover, the answers to the question of whether (or not) they have had fetishization experiences are equally distributed in all three identity groups. In general, studies on sexuality in the transgender population tend to focus more on the medical aspects of bodies and sexuality, and transfeminine individuals (see Lindley, Anzani, Prunas, & Galupo, 2020 for a critique). Including the experiences of transmasculine and nonbinary individuals allowed for an understanding that fetishization is a common experience among transgender identified people and thus merits more attention in future research.

### Limitations

This present study is the first to investigate fetishization experiences in a sample of transmasculine, transfeminine, and nonbinary individuals; however, the study is not without limitations. The participants’ responses were collected anonymously, via an online survey, in order to protect their confidentiality. This anonymity however does not allow for participants to provide any direct feedback of the study results which limits the trustworthiness of the interpretation of participants’ responses. To address this potential concern, the coding team read each participant’s response several times to ensure comprehensive understanding of our participants’ descriptions of their experiences with fetishization.

The coding of the participants’ responses was carried out by a team of cisgender researchers which could pose limitations to the appreciation of the TGNB participants’ experiences. However, the coding team sought the review of a transgender identified consultant and the supervision of the American research group to guarantee a nuanced understanding of the participants’ lived experience. Additionally, the coding team intentionally engaged in the bracketing of their cisgenderist assumptions to ensure that their interpretations did not color the experiences of the participants.

Moreover, the participants represent an online convenience sample which can overly represent the experiences of White, educated, and middle-class participants (Christian, Dillman, & Smyth, 2008). Additionally, the survey was only available in English which may have additionally limited the ability for non-White individuals to participate. Considering the current sample had limited racial diversity, with 77% of the sample identifying as White, the results should be viewed in light of this limitation. However, online sampling allowed us to reach a diverse sample in terms of gender identity. This was especially important considering previous fetishization research overlooked the experiences of transmasculine and nonbinary individuals.

Finally, the study focused on TGNB experiences with fetishization but no definition of fetishism nor fetishization was provided to the participants. On the one hand, this allowed us to grasp the positive and negative nuances of the TGNB individuals’ experiences. On the other hand, giving a univocal definition would have allowed participants to understand exactly the definition of the term and report a more connoted experience according to that shared definition.

### Implications

The present study highlighted how dehumanizing factors, such as sexual objectification and microaggressions, occur in fetishization experiences of TGNB individuals. Fear and avoidance were mentioned as some of the reactions of TGNB participants when feeling sexualized and fetishized. Avoidance of sexual and sentimental relationships due to the fear of being fetishized could be particularly problematic for TGNB individuals, as the literature suggests that sentimental and romantic relationships are an important resource and protective factor for the health of TGNB people (Galupo et al., 2019; Meier et al., 2013; Pulice-Farrow et al., 2019). From this perspective, the role of mental and sexual health professionals is to help TGNB individuals navigate the fine line between appreciation and fetishization. These dynamics have implications in terms of consent and negotiations of boundaries for TGNB individuals (Prunas, 2019). Professionals should also always keep in mind that the gender minority identity of the person may interact with other marginalized identities, in an intersectional perspective.

Sex researcher and clinicians should be cautious in labeling fetishization as a universal negative experience, and in reducing all forms of sexual and erotic attraction toward TGNB people as fetishistic. Our results show that, for some TGNC participants, being fetishized is far from a negative experience, as it is associated with enjoyment, a boost in self-esteem, or is perceived as a kinky practice. Finally, it is important to stress that the line between attraction and fetishization can be blurred in some cases, thus underlying the importance of putting the personal experiences of TGNB people at the center. Future studies may focus on broadening the perspective of observation of fetishization toward TGNB individuals, and deepen the understanding of the effects of fetishization in different contexts (i.e., interpersonal relationships vs. dating apps) and the possible short term and long term consequences to being exposed to fetishization, both in positive and negative terms.

## Appendix 1

Graphic representation of the geographical location of the respondents to the questionnaire.
